# Twin pregnancy complicated by esophageal atresia, duodenal atresia, gastric perforation, and hypoplastic left heart structures in one twin: a case report and review of the literature

**DOI:** 10.1186/s13256-016-1195-x

**Published:** 2017-03-18

**Authors:** Mohamad K. Abou Chaar, Mariana L. Meyers, Bethany D. Tucker, Henry L. Galan, Kenneth W. Liechty, Timothy M. Crombleholme, Ahmed I. Marwan

**Affiliations:** 1 Colorado Fetal Care Center, Colorado Institute of Maternal and Fetal Health, University of Colorado Denver, Anschutz Medical Center, Denver, USA; 2 Department of Pediatric Radiology, University of Colorado Denver, Anschutz Medical Center, Denver, USA; 3 Department of Pediatrics, University of Colorado Denver, Anschutz Medical Center, Denver, USA; 4 Department of Obstetrics and Gynecology – Maternal Fetal Medicine, University of Colorado Denver, Anschutz Medical Center, Denver, USA; 5 Department of Pediatric Surgery, Children’s Hospital Colorado, University of Colorado Denver, Anschutz Medical Center, Denver, USA; 6 Surgery and Pediatrics, Colorado Fetal Care Center – Children’s Hospital Colorado, 13123 East 16th Avenue, 328, Aurora, Colorado 80045 USA

**Keywords:** Monochorionic diamniotic twins, Esophageal atresia without tracheoesophageal fistula, Duodenal atresia, Gastric perforation, Fetal MRI, Case report

## Abstract

**Background:**

The antenatal diagnosis of a combined esophageal atresia without tracheoesophageal fistula and duodenal atresia with or without gastric perforation is a rare occurrence. These diagnoses are difficult and can be suspected on ultrasound by nonspecific findings including a small stomach and polyhydramnios. Fetal magnetic resonance imaging adds significant anatomical detail and can aid in the diagnosis of these complicated cases. Upon an extensive literature review, there are no reports documenting these combined findings in a twin pregnancy. Therefore we believe this is the first case report of an antenatal diagnosis of combined pure esophageal and duodenal atresia in a twin gestation.

**Case presentation:**

We present a case of a 30-year-old G1P0 white woman at 22-week gestation with a monochorionic-diamniotic twin pregnancy discordant for esophageal atresia, duodenal atresia with gastric perforation, hypoplastic left heart structures, and significant early gestation maternal polyhydramnios. In this case, fetal magnetic resonance imaging was able to depict additional findings including area of gastric wall rupture, hiatal hernia, dilation of the distal esophagus, and area of duodenal obstruction and thus facilitated the proper diagnosis. After extensive counseling at our multidisciplinary team meeting, the parents elected to proceed with radiofrequency ablation of the anomalous twin to maximize the survival of the normal co-twin. The procedure was performed successfully with complete cessation of flow in the umbilical artery and complete cardiac standstill in the anomalous twin with no detrimental effects on the healthy co-twin.

**Conclusions:**

Prenatal diagnosis of complex anomalies in twin pregnancies constitutes a multitude of ethical, religious, and cultural factors that come into play in the management of these cases. Fetal magnetic resonance imaging provides detailed valuable information that can assist in management options including possible prenatal intervention. The combination of a cystic structure with peristalsis-like movement above the diaphragm (for example, “the upper thoracic pouch sign”), polyhydramnios, and progressive distention of the stomach and duodenum should increase suspicion for a combined pure esophageal and duodenal atresia.

## Background

The incidence of congenital anomalies in twin gestation is as high as 6 % [1]. Furthermore, the associated risk of prematurity when counseling parents with anomalous twin gestations is an important factor since the average gestational age at delivery for anomalous twins is 35.7 weeks [2]. Congenital anomalies in twins vary considerably and may affect one or a combination of organ systems. Uncommon gastrointestinal anomalies include esophageal atresia (EA), tracheoesophageal atresia, and duodenal atresia (DA). The prenatal diagnosis of EA without tracheoesophageal fistula (TEF) can be difficult to make; it is typically first suspected on ultrasound (US) with the findings of a small or absent fetal stomach bubble, an upper thoracic pouch sign that signifies an additional cystic structure in the chest representing the distal esophagus, and associated polyhydramnios [3, 4]. The diagnosis by US, however, is challenging and magnetic resonance imaging (MRI) may add additional details to solidify the diagnosis. Once the prenatal diagnosis of an EA is made, a thorough search for other associated defects should be pursued since it is known that 50 % of cases of EA are found to have other congenital anomalies including vertebral anomalies, anal atresia, cardiac defects, tracheoesophageal fistula and/or esophageal atresia, renal anomalies, and limb abnormalities (VACTERL), coloboma, heart defect, choanal atresia, retarded growth, genital abnormality and ear abnormality (CHARGE) syndrome, or chromosomal anomalies [5]. The overall incidence of EA is 1 in 4099 births worldwide [6] and only 7 % of these cases are without TEF [5]. Compared to EA, DA occurs in approximately 1 in 6000 to 10,000 live births and is associated with an approximately 5 % mortality and long-term complications [7]. Concomitant DA and EA is very rare, and twin cases detected prenatally have not been reported in the English literature. To the best of our knowledge, a review of the published literature suggests that this is the first case of a discordant monochorionic-diamniotic twin pregnancy that presented with DA and pure EA (Table 1).

**Table 1:** Ultrasound and magnetic resonance imaging associated findings, and fetal karyotype in reported cases with prenatally diagnosed esophageal atresia with duodenal atresia

Authors; year	GA at Dx	Associated ultrasound and magnetic resonance imaging findings	Karyotype
Duenhoelter *et al*. [31] 1976	Near term	Ultrasound showed two communicating upper abdominal masses. It also failed to ingest contrast media after injection of radiopaque material into the amniotic sac	Normal
Hayden *et al*. [8] 1983	34	Ultrasound showed two large fluid-filled cystic structures within the fetal abdomen	T21
Estroff *et al*. [32] 1994	16.5	Ultrasound showed an abdominal C-shaped fluid collection suggesting a dilated stomach which extended into the chest behind the heart. A follow-up ultrasound showed continuity between the fluid collection in the fetal chest and the dilated stomach. Later, fetal ascites and skin edema developed	Normal
	16.7	Ultrasound showed an abdominal C-shaped fluid collection suggesting a dilated stomach with progressive polyhydramnios	Normal
	22.5	Ultrasound showed an abnormal triangular cranial shape in addition to dumbbell-shaped stomach. Mild to moderate polyhydramnios was appreciated	Normal
Pameijer *et al*. [33] 2000	18	Level II ultrasound showed a cystic mass, which was assumed to be the stomach, with a posterior mediastinal, intrathoracic portion compressing the left atrium. No evidence of dilated proximal esophageal pouch. On follow-up polyhydramnios was appreciated with the presence of the classic “double bubble” sign. Ultrafast fetal magnetic resonance imaging showed a cystic mass extending through the esophageal hiatus. Postnatal findings: pure esophageal atresia, duodenal atresia, biliary atresia, and pancreatic ductal atresia. Subsequently, the neonate was diagnosed with a type I choledochal cyst	T21
Marquette *et al*. [34] 2004	12	Ultrasound showed a single large cystic structure in the anterior upper abdomen	T21
Mitani *et al*. [11] 2009	25	Ultrasound showed a double cystic structure with a dilated stomach and duodenum and an intrathoracic cyst. Magnetic resonance imaging and ultrasound at 26 weeks showed shrinkage of the stomach and duodenum and appearance of massive ascites, suggesting rupture of either structures. Distention of the proximal esophagus was noted as an “upper pouch” sign. Polyhydramnios was noted at 30 weeks	Normal
Kanasugi *et al*. [24] 2013	20	Ultrasound showed multicystic dysplastic left kidney, normal-sized stomach, no abdominal cysts, no cardiac anomalies, and no limb abnormalities. At 31 weeks of gestation, fetus A developed a left hydroureter with dilatation of the colon	Normal
Kadohira *et al*. [25] 2014	17	Ultrasound showed intra-abdominal cystic mass with typical “double bubble” sign with no other structural abnormalities. Follow-up ultrasound at 26 weeks demonstrated marked dilatation of the stomach and duodenum. Similar to our findings, a peristalsis-like movement was appreciated in the mediastinum of the fetus and subsequently confirmed by magnetic resonance imaging to be a dilated distal esophageal pouch	Normal

*Dx* diagnosis, GA *gestational age*

## Case presentation

**Fig. 1:**
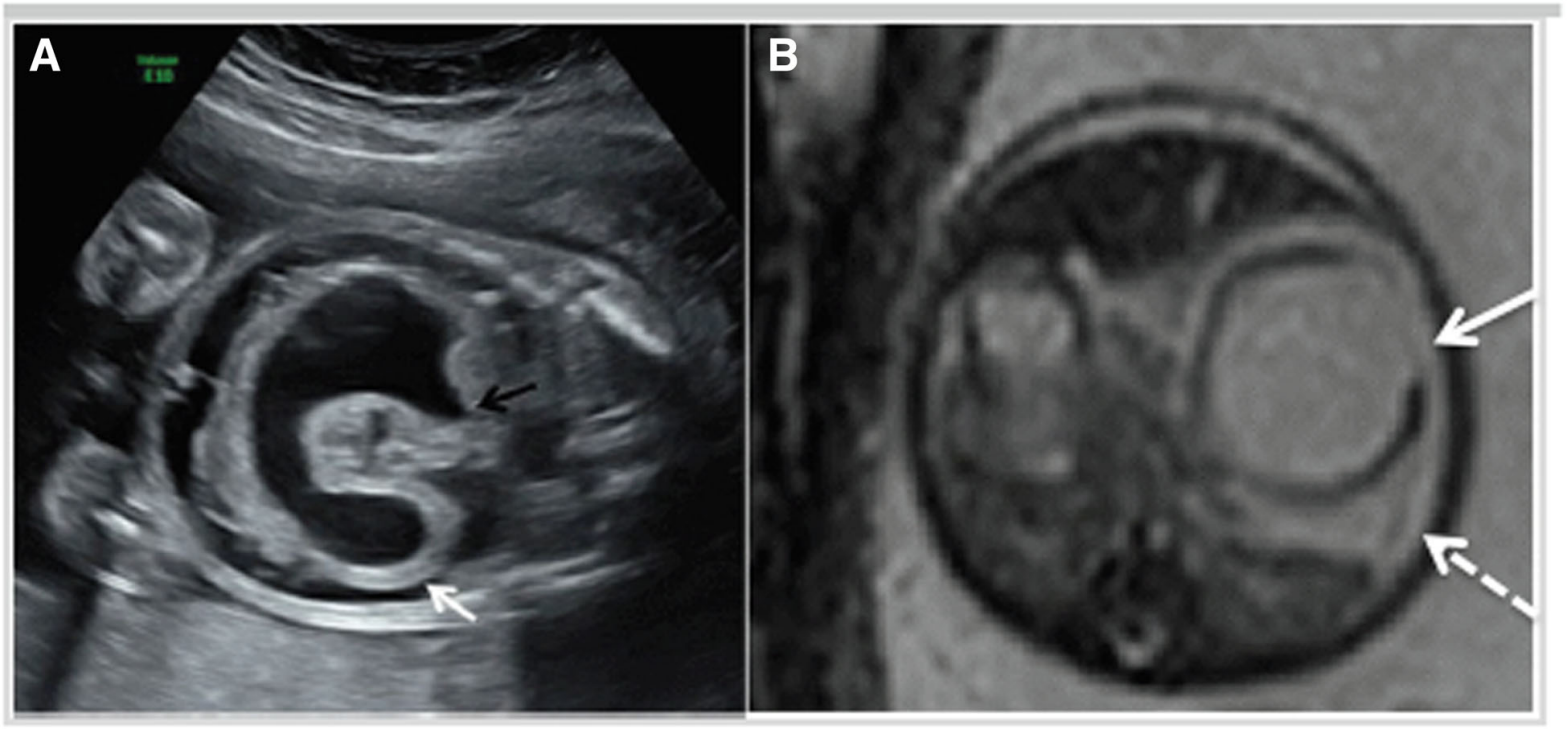
**a** Transverse ultrasound image of twin B showing the dilated and thick-walled stomach with a “cut-off” at the second portion of the duodenum (*white arrow*). *Black arrow* shows the area of fluctuation of the gastroesophageal junction. **b** Axial T2-weighted magnetic resonance image of the twin pregnancy showing the dilated fluid-filled stomach with evidence of wall rupture (*white arrow*) and subsequence ascites (*dashed arrow*)

**Fig. 2:**
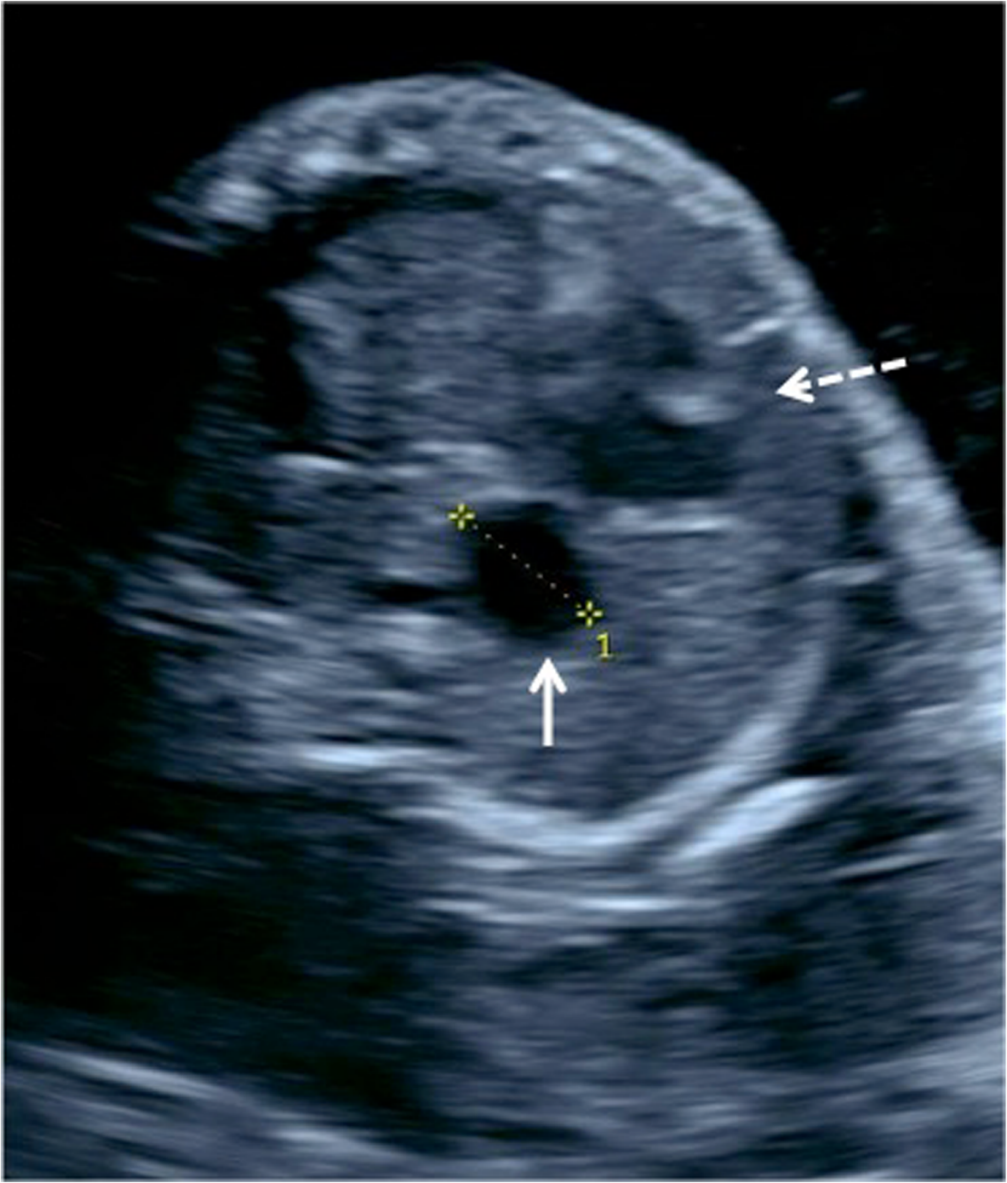
Transverse ultrasound view of the fetal chest showing the cystic structure (*white arrow*) posterior to the heart (*dashed arrow*)

**Fig. 3:**
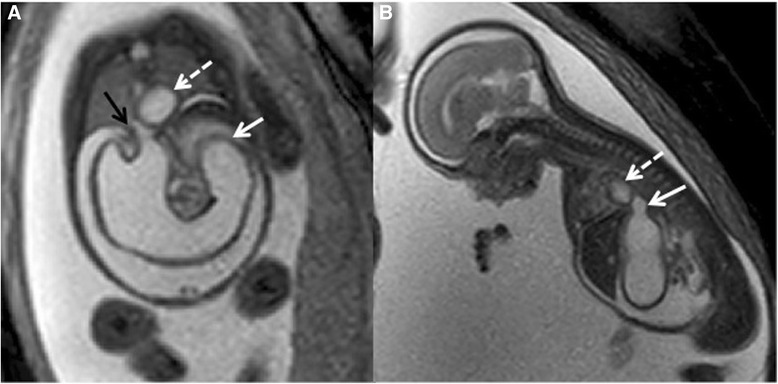
**a** Coronal T2-weighted magnetic resonance image shows a dilated stomach with cut-off at the second–third duodenal segment (*solid white arrow*), fluid-filled hiatal hernia (*black arrow*), and the dilated distal esophagus (*dashed arrow*) correlating with the cystic structure posterior to the heart seen on the ultrasound. **b** Sagittal T2-weighted magnetic resonance image shows dilated stomach with fluid dilatation at the gastroesophageal junction consistent with a hiatal hernia (*solid white arrow*). The fluctuating cystic structure posterior to the heart is also seen (*dashed arrow*); it is thought to represent the distal esophagus

A 30-year-old primigravida (G1P0) white woman was referred to the Colorado Fetal Care Center (CFCC) at 22 weeks and 5 days’ gestation following a 16-week US obtained at another facility that demonstrated monochorionic-diamniotic twin pregnancy with signs of duodenal obstruction, polyhydramnios, and excess fluid in the esophagus of twin B. Her medical history was negative for hypertension and diabetes, and negative for thyroid, heart, lung, or kidney disease. There was no prior blood transfusion, hepatitis, or urinary tract infection. She denied any significant exposures to tobacco, alcohol, drugs, radiation, or hazardous chemicals during the pregnancy. Her surgical history was negative; she denied any congenital anomalies or chronic conditions. The same is true for the father of the pregnancy. She was taking prenatal vitamins but no other medications. A dual sac amniocentesis was performed yielding a 46,XY karyotype and normal chromosomal microarray analysis (CMA) results for each twin. An obstetrical US at CFCC revealed normal cervical length and normal growth and anatomy for twin A. Twin B, however, showed polyhydramnios with a deepest vertical pocket (DVP) of 11.1 cm, markedly enlarged stomach with thickened echogenic walls, and moderate ascites (Fig. 1a, b), and a cystic structure posterior to the heart (Fig. 2). Ultrafast fetal magnetic resonance imaging (MRI) demonstrated a dilated lower esophagus that fluctuated in size on successive images over the course of the examination (Fig. 2). Furthermore, a hiatal hernia was also noted which also fluctuated in size (Fig. 3a, b). The MRI confirmed the US findings of ascites; however, it provided detailed anatomy to diagnose gastric perforation by identifying rupture of the gastric wall (Fig. 1b). Although antenatal cases of DA usually manifest with a large stomach bubble, gastric distention in combined pure EA and DA is more significant than in DA alone, which is attributed to the accumulation of secretions within this closed loop [8]. The first duodenal segment was dilated with an abrupt cut-off at the level of the second–third segments (shown in Fig. 3a). Fetal echocardiography demonstrated small left heart structures, hypoplastic aortic arch with intermittent flow reversal in the aortic isthmus, and persistent left superior vena cava (SVC) to a dilated coronary sinus with possible coarctation of the aorta. Similar to the US findings, a large fluid-filled structure was identified posterior to the heart causing a mild mass effect on the left atrium.

Our patient and her husband were counseled at a multidisciplinary family team meeting including review of the pertinent images, explanation of the development and natural history of all findings, discussion of the surgical management of the various anomalies, and possible management options for the pregnancy. The complex nature of the anomalous twin’s case was explained in detail regarding the complicated EA repair in the setting of DA and gastric perforation and the possible need for multiple-stage repairs of the hypoplastic left heart anatomy. The family was counseled regarding various management options including: (1) continuation of pregnancy with possible amnioreduction for symptomatic relief, (2) pregnancy interruption, (3) comfort care at delivery, and (4) selective reduction of pregnancy using radio frequency ablation with amnioreduction. They opted to proceed with selective reduction. After obtaining an informed consent, an US-guided radiofrequency ablation (RFA) of the cord of the anomalous twin was performed at 23 weeks’ gestation using 100 watts of power targeting the abdominal cord insertion site [9]. Two treatments were applied with confirmation of complete cessation of blood flow in the cord with no cardiac activity. Throughout the procedure the normal co-twin had a reassuring fetal heart rate and middle cerebral artery Doppler evaluation. Our patient had an uneventful continuation of her pregnancy. At 37 weeks 4 days she went into spontaneous labor. The fetus demonstrated some variable decelerations prompting a vacuum-assisted delivery. She gave birth to a healthy 3.4 kg baby with Appearance, Pulse, Grimace, Activity, and Respiration (APGAR) scores of 8 and 9. A placental biopsy revealed a 522 gm placenta fused disc that was greater than the tenth percentile for gestation with unequal shares favoring twin A (80/20). Twin A had a three-vessel cord with no abnormality and twin B had a two-vessel cord with marginal insertion. The villus tissue of twin B demonstrated infarction and degeneration. No autopsy of Twin B was performed due to the interval between RFA and delivery, and the remains were cremated precluding further genetic studies. At the time of submission of this manuscript, the neonate is 9-months old and doing very well with no concerns.

## Discussion

Monozygotic twinning accounts for 30 % of all twin pregnancies and is usually associated with a higher risk of complications including: prenatal mortality, preterm delivery, intrauterine growth restriction (IUGR), and congenital anomalies. The breadth of anomalies is extensive. Midline structural defects are more common in monozygotic twinning including, but not limited to, conjoined twins, acardiac fetus, sacrococcygeal teratoma, cloacal exstrophy, sirenomelia, neural tube defects, holoprosencephaly, anal atresia, and TEF [1]. The early disturbance in organogenesis that results in EA deformities also affects other organ systems. These associated anomalies are most commonly seen in pure EA without TEF and can potentially significantly alter the treatment strategy and affect survival and long-term outcomes [10]. Numerous reports have suggested that between 50 and 70 % of babies with EA have at least one associated congenital malformation. The association of pure EA (gross type A) and DA is very rare. There have been a handful of cases of prenatally diagnosed combined EA with DA reported in the English literature [11]. Our report contributes an additional case that is unusual in that the diagnosis is made in a monochorionic-diamniotic pregnancy. 

Prenatal diagnosis of EA without TEF is difficult with a false positive rate of 78 % [12]. There are several published case reports in singleton pregnancies that highlight the features and the variability seen in EA anomalies. A recent review by Ethun *et al*. [13] reported that polyhydramnios has the highest sensitivity with regards to prenatal diagnosis of EA; however, since it is associated with a variety of other conditions, it carries a low specificity. Furthermore, a small or absent stomach bubble might not be present with concomitant DA; in which case, an enlarged stomach will be detected, giving the two previous findings a combined 85 % positive predictive value. Finally, they concluded that the presence of an upper pouch sign indicating dilatation of the blind-ending upper esophagus has a 100 % positive predictive value [13]. DA is usually prenatally diagnosed by the presence of the classic double bubble sign on US and/or fetal MRI.

DA is associated with a high incidence of trisomy 21. When DA is detected prenatally, many sources suggest the incidence of trisomy 21 is around 30 %, but some authors have reported incidences up to 41 % and 46 % [14–16]. Likewise, the incidence of chromosome anomalies is increased in EA, with an incidence of 6 to 10 % for whole chromosome aneuploidy and an additional 1 to 2 % for copy number variants [17]. Single gene deletions, such as *SHH* and *GLI3*, have previously been implicated in animal models of EA, and more recently reported in human cases [18–20]. Therefore, it is appropriate to offer prenatal diagnosis, which aids in the management of pregnancy and informs postnatal care of the neonate. Since 2013, the American College of Obstetricians and Gynecologists (ACOG) has recommended CMA for pregnancies with a fetal anomaly [21, 22]. CMA can detect copy number variants, but it cannot detect single gene disorders such as Feingold syndrome, which is an autosomal dominant condition due to mutations in the *MYCN* gene characterized by EAs, DAs, microcephaly, intellectual disability, and digital anomalies [23]. Other single gene disorders have been associated with EA or DA, but these two conditions are rarely seen together. In addition to trisomy 21, DA is associated with cardiac defects, annular pancreas, malrotation, anorectal atresia, and genitourinary anomalies [14]. Thus, when DA is detected prenatally a detailed anatomic scan is necessary to look for associated anomalies. In the setting of concomitant defects, the stomach will be filled with secretions to a much greater extent than what is seen in DA alone, which results in an enormously dilated stomach and proximal duodenum with possible antecedent perforation. Such massive accumulation of secretions within this closed loop, extending from the distal esophagus, stomach, and proximal duodenum, has been considered to be pathognomonic by Hayden *et al*. [8].

A very interesting case was recently reported by Kanasugi *et al*. [24] at 20 weeks’ gestation, where a monochorionic-diamniotic twin pregnancy, a product of intracytoplasmic sperm injection, demonstrated a multicystic dysplastic left kidney in one of the twins. At 31 weeks, a left hydroureter was diagnosed along with appreciation of a dilated colon. However, neither an amniotic fluid volume abnormality nor a prenatal reference to EA was made. The twins were delivered via C-section at 36 weeks due to premature rupture of membranes (PROM). The anomalous twin was further examined and found to have hypospadias, anorectal malformation, type C EA, and DA. The renal anomalies that were diagnosed prenatally were confirmed with US establishing the diagnosis of VACTERL association in that twin. In stark contrast to this report, the affected anomalous twin in our case demonstrated a prenatal diagnosis of a pure EA, no renal anomalies, and the pregnancy was the result of natural conception [24]. Furthermore, Kadohira *et al.* [25] recently reported a fetus with normal karyotype at 17 weeks’ gestation with an intra-abdominal cystic mass. Follow-up US at 26 weeks demonstrated marked dilatation of the stomach and duodenum. Similar to our findings, a peristalsis-like movement was appreciated in the mediastinum of the fetus and subsequently confirmed by MRI to be a dilated distal esophageal pouch. In their case, Kadohira *et al*. [25] reported a marked increase in the fetal abdomen size in the third trimester and utilized the novel idea of repeat fetal stomach paracentesis, to successfully avoid gastric perforation. To the best of our knowledge, this maneuver has not been utilized in twin gestations [25].

Our case demonstrates that MRI and US are complementary to each other. It was by US that the initial abnormalities were identified, which prompted further evaluation; emphasizing that US is the first line of diagnosis. However, MRI was able to depict additional findings (area of gastric wall rupture, hiatal hernia, dilation of the distal esophagus, and area of duodenal obstruction) suggesting the underlying pathophysiologic process that, in turn, helped the parents make an informed decision regarding the treatment options.

A monochorionic placenta has various vascular anastomoses that makes it the culprit for twin-to-twin transfusion syndrome (TTTS), and facilitates intrauterine fetal transfusions that may occur with the *in utero* fetal demise (IUFD) of one twin. With IUFD ensuing, the surviving co-twin might have neurological deficits caused by acute severe hypotension and the resultant brain hypoperfusion that is caused by the shunting of blood via anastomoses to the dying twin [26, 27]. One approach to managing these complicated anomalous twin pregnancies is to attempt separation of the fates of the twins either utilizing laser photocoagulation or selective reduction. Management of these complicated pregnancies constitutes a moral and ethical dilemma for both the parents and physicians involved. While selective termination may be rarely pursued in the setting of minor malformations, it is certainly a viable option in severe anomalies provided it aligns with the social, moral, and religious beliefs of the parents. One limitation of our report is that there was no autopsy performed. In our case, after counseling and management discussions, the parents elected to proceed with selective reduction via RFA in order to maximize the survival benefit of the normal co-twin [27, 28]. More specifically, the procedure was performed to avoid the risks associated with the demise of an anomalous co-twin (for example, risk of double demise or neurologic injury to surviving normal twin) and to gain the secondary benefit of maturity with delivery later in gestation. Since the twins were monochorionic they were presumed to be monozygous, so no significant variation in the genetic makeup of the affected and unaffected twins would be expected. In fact, their karyotypes and chromosomal microarray analyses were both normal, with no sign of discordance. In 2012, Veenma and colleagues were unable to find evidence of differences in copy number variations (CNVs) as the cause of twin discordance for EA [28, 29]. Moreover, phenotypic discordance has been reported in monozygous twins with cell lines that were discordant for trisomy 21 and monosomy X. In addition, cases of monozygous twins discordant for single gene disorders, such as neurofibromatosis and Dravet syndrome, have also been reported [29, 30]. Thus far, however, cases of twins discordant for EA or DA and single gene mutations have not been reported. Alas, no additional testing could be performed for this case.

## Conclusions

Upon review of the literature, this is the first report of prenatal diagnosis of both pure EA and DA findings in one fetus of a twin gestation. Concomitant pure EA and DA is a rare occurrence. The presence of a cystic structure with peristalsis-like movement above the diaphragm (for example, “the upper thoracic pouch sign”), polyhydramnios, and progressive distention of the stomach and duodenum, provide a high index of suspicion of a combined pure EA and DA. Understanding the variety of abnormalities associated with EA and DA in the prenatal period and understanding the salient US and MRI characteristics of these two structural abnormalities alone and in combination, are invaluable in the counseling and management options for patients. Given that selective reduction of anomalous fetuses is a valid management option, especially to maximize co-twin survival in monochorionic-diamniotic twin gestation, a reasonably firm diagnosis based on imaging modalities is necessary as autopsy is not an option in reduced pregnancies.

## Abbreviations

ACOG: American College of Obstetricians and Gynecologists
APGAR: Appearance, Pulse, Grimace, Activity, and Respiration
CFCC: Colorado Fetal Care Center
CMA: Chromosomal microarray analysis
CNV: Copy number variation
DA: Duodenal atresia
DVP: Deepest vertical pocket
EA: Esophageal atresia
IUFD: *In utero* fetal demise
IUGR: Intrauterine growth restriction
MRI: Magnetic resonance imaging
PROM: Premature rupture of membranes
RFA: Radiofrequency ablation
SVC: Superior vena cava
TEF: Tracheoesophageal fistula
TTTS: Twin-to-twin transfusion syndrome
US: Ultrasound
VACTERL: Vertebral anomalies, anal atresia, cardiac defects, tracheoesophageal fistula and/or esophageal atresia, renal anomalies, and limb abnormalities
